# Supplementary material for: Systematic review of electron transfer study in DNA relevant to Parkinson’s disease and scanning tunneling microscopy
Source: PeerJ. 2025 Aug 20;13:e19807. doi: 10.7717/peerj.19807 (PMC12374687; doi:10.7717/peerj.19807)
Supplement: Supplemental Information 3 [file peerj-13-19807-s003.docx]

**Table S1 Comparison of experimental and review articles in previous studies, including study design, techniques, samples/models, and findings regarding electron transfer, DNA, and PD.**

| **Author, article type** | **Study design** | **Techniques** | **Samples/ models** | **Comments on findings** |
| --- | --- | --- | --- | --- |
| Abha *et al.* (2020)  Experimental | Synthesis of bovine serum albumin (BSA)-capped manganese-doped zinc sulphide quantum dots (Mn:ZnS/BSA QDs) using a chemical co-precipitation method to detect dopamine (DA). | Mn:ZnS/BSA ultraviolet-visible adsorption spectroscopy; Photoluminescence spectroscopy; Atomic force microscopy. | DA in human serum and human urine samples. | The proposed method has been reliably used for the detection of DA in real samples. The good recovery percentage between 96 and 104 was obtained for urine and serum analysis. |
| Atta *et al.* (2019) Experimental | Modification of the gold electrode surface with Nafion, β-cyclodextrin, and gold nanoparticles to determine DA in biological fluids. | Electrochemical sensing (cyclic voltammetry); Scanning electron microscopy. | DA in human urine samples. | The concurrent identification of DA, ascorbic acid (AA), and uric acid (UA) holds great significance in the domains of diagnostic and pathological research, neurochemistry, and biomedical chemistry. The proposed approach can distinguish between DA and other substances such as AA, UA, acetaminophen, serotonin, and tyrosine in biological fluids. |
| Benecke *et al.* (1993)  Experimental | Measurements of electron transfer complexes (ETCs) I, III, and IV activities from idiopathic Parkinson’s disease (PD) and Parkinson-plus syndromes patients. | Assays of ETCs activities were performed at 30°C using a dual-wavelength spectrometer. | Human blood. | Repeated measurements in five PD patients in the early stages shown a significant drop in the activity of complex I and IV within a year. No differences were seen between the control individuals and PD patients, except in the substantia nigra, SN. This suggests that complex I deficiency is specifically localized to the SN. |
| Benzi and Moretti (1995) Review | Review of the possible contribution of mitochondrial processes to forming and releasing reactive oxygen species (ROS) in the aging brain and the age-related changes in antioxidant defenses. | Biochemical and histochemical studies in cultured cells. | Rodents brain: Wistar, Sprague-Dawley rats; C57B1 mice; Gerbils. | Elevated production and discharge of ROS exacerbate impairment of mitochondrial activity and other cellular components. The process of aging has been linked to changes in the composition and functioning of cell membranes, increased oxidation of proteins, and an elevated occurrence of oxidative damage and mutations in mitochondrial DNA. |
| Camargo *et al.* (2018) Review | Review of advantages and drawbacks of adding different electron transport branches into mammalian and insect mitochondria. | Biochemistry in expression of the alternative oxidase. | *Drosophila melanogaster* or fruit fly. | Benefits and risks of introducing transgenic expression of alternative enzymes that provide electron transfer pathways additional to the oxidative phosphorylation system. |
| Carreras *et al.* (2004) Review | Review of association of nitric oxide (NO) leading to genetic or acquired NO-mediated damage in mitochondrial complex I. | Biochemical analysis of mitochondrial protein. | Mitochondria of rat skeletal muscle, liver, and heart. | Complex I is very sensitive to hereditary or acquired NO-mediated injury. Increased O_2_^-^ generation by complex I prolongs organ damage in common diseases through energy failure, oxidative-nitrosative stress, and by redirecting normal cell communication to apoptosis-inducing pathways. |
| Chatterjee *et al.* (2019) Experimental | An application of new hybrid ligand (SCG – full name not mentioned) fluorescence to study and detect the early events of the folding/aggregation kinetics of α-synuclein, a protein involved in the pathology of PD. | Fluorescence correlation spectroscopy. | Cultures of SH-SY5Y cells. | The synthesized hybrid ligand, known as the fluorescent probe SCG, has the ability to specifically identify cysteine in either the conformational or redox states of cysteine residues within a protein. This detection process takes about three minutes, making it a practical and highly sensitive method for monitoring the initial stages of α-synuclein aggregation. |
| Diaz-Diestra *et al.* (2017) Experimental | Synthesis of room-temperature phosphorescence sensor based on L-cysteine-capped manganese-doped zinc sulphide quantum dots (L-cys ZnS:Mn QDs) to detect DA in biological fluids in a wide range of concentrations (nM – µM). | High-resolution transmission electron microscopy; L-cys ZnS:Mn QDs using phosphorescence spectroscopy. | Human urine samples. | The developed sensor is capable of detecting DA even at low concentrations in biological fluids, such as cerebrospinal fluid, which is commonly associated with neurodegenerative disorders like PD. This sensor is highly sensitive within the nanomolar range, with a limit of detection of approximately 8 nM, and it consistently produces accurate results. |
| Ehlich and Hörber (2009) Experimental | Investigation of the electron transport through the synthesized nanostructure, which is nanodots, functionalized with single-stranded DNA (ssDNA) molecules. | Scanning tunneling microscopy (STM). | 50-base and 12-base of ssDNA bound to gold (Au dots and Au (111)). | Nanodots are promising candidates for efficient charge generation by light absorption, enabling chemical reactions to occur at the dots, hence facilitating light energy storage. The disparity in molecular arrangement between each film elucidates the reason behind the diminished conductivity of the ssDNA layer on the Au dot in comparison to the layer on Au (111), which exhibits enhanced conductivity. |
| Fato *et al.* (2008) Review | Emphasis on some novel findings on the NADH coenzyme Q oxidoreductase enzyme that are of relevance to the pathogenesis of neurodegenerative diseases. | Biochemical analysis of mitochondrial protein. | Rat heart mitochondria. | Primary mitochondrial malfunction may result in increased ROS generation, initiating cell death processes in dopaminergic cells via an interplay of endogenous and external stimuli. In fact, parkinsonism-inducing neurotoxins such as 1-methyl-4-phenylpyridinium ion (MPP+) and rotenone enhance ROS production in complex I. |
| Greenamyre *et al.* (2001) Review | Biochemical studies of complex I mammalian electron transport chain to determine the presence of defect in complex I of PD caused by rotenone. | Spectrophotometric assay; Mitochondrial respiration assay; Autoradiographic assay. | Human brain specimens; Brain, heart, liver, and kidney specimens of rats. | The inhibition of complex I by rotenone uniformly affected the entire brain, resulting in the replication of the clinical, biochemical, and behavioral characteristics of PD. |
| Guarani *et al.* (2014) Experimental | Interaction proteomics-based analysis to interrogate the molecular associations of 15 core subunits and assembly factors previously linked to human complex I deficiency. | Quantitative proteomics of complex I; Complex I activity dipstick enzyme assay. | Cultures of HCT116, HeLa, and HEK293T cells. | Depletion of TIMMDC1, a protein with a projected structure of four transmembrane segments, led to a decrease in the activity of complex I and cellular respiration. The diminished complex I activity has been associated with neurodegenerative conditions, such as PD. |
| Hashimoto *et al.* (1999) Experimental | Investigation of heme protein cytochrome c in α-synuclein aggregation leading to PD. | Aggregation assays *in vitro*; Immunoblot analysis; Electron microscopy; Immunohistochemistry. | Human α-synuclein and bovine heart cytochrome c proteins. | Cytochrome c, a widely recognized molecule responsible for transferring electrons in mitochondria and a facilitator of cell death through apoptosis, may play a role in the aggregation of α-synuclein in PD and associated illnesses, which is triggered by oxidative stress. |
| Imai (2020) Review | Suppression of the PINK1 and Parkin genes to investigate the aberrant wing posture and mitochondrial deterioration. | Wing phenotype study. | Drosophila (fruit fly);  Human brain tissue. | Screening for modifiers with the Drosophila wing phenotype showed that turning off Miro, a mitochondrial adaptor protein, reduces the effect of mitochondrial degeneration. |
| Jiang *et al.* (2016) Experimental | Investigation into the impact of compound A on the succinate dehydrogenase subunit B (SDHB) protein, a component of complex II in the mitochondrial electron transport chain. Compound A effectively obstructs Bim-induced apoptosis by creating pores in the outer membrane of the mitochondria after Bax activation. | Immunoblot analysis (western blotting). | 6-hydroxydopamine (6-OHDA)-induced PD model in rats. | Compound A, which specifically targets the SDHB protein of complex II in the mitochondrial electron transport chain, substantially inhibits Bim-driven apoptosis when Bax is activated on the mitochondria in a PD model induced by the dopamine derivative 6-OHDA. Compound A preserves the integrity of the electron transport chain and allows the treated cells to continue multiplying despite the initiation of programmed cell death (apoptosis). The chemical inhibited the death of dopaminergic neurons and successfully corrected Parkinson-like behavior in the rat model of PD. |
| Konijnenberg *et al.* (2016) Experimental | Investigation of the preliminary stage of α-synuclein monomer interaction with epigallocatechin-3-gallate (EGCG), and DA to better understand the molecular action of modulators of α-synuclein aggregation. | Nanoelectrospray-ionization mass spectrometry; Ion mobility; Native top-down electron transfer dissociation. | EGCG, DA and wild-type human α-synuclein. | DA-induced α-synuclein oligomers exhibit cytotoxicity in cultured cells and have been shown to form elongated protein−ligand complexes. The α-synuclein oligomers generated by EGCG form tight protein−ligand complexes that do not harm cultured cells. The various effects on the α-synuclein aggregation mechanism are likely due to the presence of different ligands. |
| Kuter *et al.* (2019) Experimental | The impact of mitochondrial electron transport chain in complexes I, II, and IV, as well as their higher assembled forms known as supercomplexes, on the functioning of dopaminergic neurons that have degenerated due to the selective toxin 6-OHDA and/or the death of 30% of astrocytes caused by the chronic infusion of the glial toxin fluorocitrate in the rat SN. | Inducing chronic infusion of the glial toxin fluorocitrate on functioning of the mitochondrial ETCs I, II, IV and their higher assembled forms, supercomplexes in the rat SN. | Animal model of early PD (3-month old male Wistar Han rats). | The enhanced operation of the electron transfer complex, particularly complex I and IV, is crucial in the compensatory response of the dopaminergic system. The surviving neurons exhibit increased energy demands to offset the loss of a fraction of the dopaminergic neurons. |
| La *et al.* (2014) Experimental | Investigation on the effect of the presence of iron ligation of nitrilotriacetic acid (NTA), a well-known tetradentate ligand, on the catalytic oxidation reaction of DA. | Electrochemistry; Ultraviolet-visible spectroscopy. | Synthetic molecules of DA-Fe(III) and DA-Fe(III)-NTA. | The total occupation of all binding sites of Fe(III) by DA, along with the subsequent attachment of NTA, was observed to entirely counteract the catalytic influence of Fe(III) on the oxidation of DA. The electron transport from O_2_ to DA is hindered due to the inability of O_2_ to access the metal core, resulting in the Fe(III) bridging being hampered. |
| Lee *et al.* (2005) Experimental | Elucidation of α-synuclein conformational dynamics in the aggregation process that is implicated in the pathogenesis of PD. | Fluorescence energy transfer (FET) kinetics measurements. | α-Synuclein protein. | This experiment discusses on the α-synuclein protein exhibiting a range of different conformations at physiological pH, with a significant proportion of these conformations being in the form of extended structures. |
| Lee *et al.* (2007) Experimental | Comparison of measured and calculated contact rates in α-synuclein. | FET kinetics measurements. | Site-directed mutagenesis of α-synuclein proteins. | The measured contact rate constants are in agreement with the estimated rates, providing additional evidence of the high dynamism of the polypeptide. Alterations in these dynamics as the α-synuclein protein undergoes aging and aggregation could elucidate the mechanism of plaque development, a process hypothesized to be responsible for PD. |
| McEwen *et al.* (2009) Experimental | The study of electron transfer characteristics of synthetic DNA sequence from pathogenic *Cryptosporidium parvum,* self-assembled on a gold surface. | Electrochemistry and STM. | Synthetic DNA on gold surface. | No relation to PD study. However, the study focuses on using STM to investigate the process of electron transfer in synthetic DNA derived from *C. parvum*. This particular organism is responsible for causing cryptosporidiosis, a parasitic disease that affects the intestinal tract of mammals. The main symptoms of cryptosporidiosis include sudden, watery, and nonblood diarrhea. |
| Mizuno *et al.* (1994) Review | Review study on the pathogenesis of nigral cell death in PD focusing mainly on studies in the Japanese population. | Study of loss of alpha-ketoglutarate dehydrogenase complex (α-KGDHC) by immunohistochemistry; Study of loss of complex I using western blotting. | Human brain mitochondria from post-mortem; Nigral and striatal co-culture from rat embryo. | While exposure to an environmental neurotoxic substance may not be the sole determinant in the development of PD, it is important to consider that hereditary predisposition also plays a vital role. |
| Mizuno *et al.* (1995) Review | Review of roles of complex I of the mitochondrial electron transfer complex and α-KGDHC in the SN of patients with PD. | Western blotting; Immunohistochemistry. | Autopsy human brain tissue; Mouse brain mitochondria. | The primary cause of cell death in the SN in PD is an energy crisis, which disrupts electron transport and ATP generation due to the simultaneous loss of both complex I and α-KGDHC. |
| Morikawa *et al.* (1998) Experimental | Use of various tetrahydroisoquinoline (TIQ) derivatives to investigate the toxic effects on mitochondrial respiration and the electron transfer complexes. | Measurement of oxygen consumption by polarography and enzyme activities of complexes I, II, III, and IV in mitochondria. | Mitochondrial suspensions from brains of 3-month-old male C57BL mice. | TIQ chemicals exhibit high inhibitory effects on the activity of complex I, resulting in the failure of mitochondria. This mechanism is analogous to how causes specific degeneration of the nigral region, contributing to the development of PD. |
| Navarro and Boveris (2010) Review | The study of the enzymatic activity of complexes I and IV in the brain mitochondria of old animals. | Biochemical analysis of mitochondrial protein. | Frontal cortex, hippocampus, and whole brain of rat; Rat liver mitochondria; Mouse heart mitochondria; Human brain cortex. | In the course of aging, the dysfunctions of electron transport in complexes I and IV, and enzymatic activity, in addition to the mitochondrial oxidative damage, cause the brain mitochondria to become impaired and fail to supply energy. |
| Naydenov *et al.* (2010) Experimental | Investigation of levodopa prolonged treatment of PD which leads to disabling side effects of dyskinesias. | Real-time polymerase chain reaction and gene expression microarrays. | Postmortem samples of the male human putamen from coronal slice of the brain; Culture of rat striatal neurons. | The involvement of bioenergetic function, which is influenced by the susceptibility of mitochondria, in the putamen is significant in the development of dyskinesias in PD. |
| Núñez *et al.* (2012) Review | Review of the components of iron homeostasis in neurons and on the mechanisms by which iron homeostasis is lost in PD, Alzheimer’s disease and other diseases showing iron accumulation. | Biochemical analysis of mitochondrial protein. | Mouse brain; Rat brain. | Age-related iron accumulation makes neurons in brain regions susceptible to degeneration more vulnerable to oxidative stress, which causes cellular damage. |
| Pastoris *et al.* (1995)  Experimental | Exploitation of the neurotoxic effects of 1-methyl-4-phenyl-1,2,3,6-tetrahydropyridine (MPTP) in primates to examine the physiopathology of PD, which may potentially result in skeletal muscle functioning changes. | Biochemical assays: Enzyme activities related to energy transduction in skeletal muscles were evaluated from MPTP-treated monkeys. | Gastrocnemius, soleus, and biceps muscles from *Macaca pascicularis* (monkey). | MPTP inhibited the enzymatic activity linked to the Krebs cycle and the electron transport chain in the biceps muscles. The hypothesis suggests that the Parkinson-like condition induced by MPP+ is linked to the decrease in activity of cerebral mitochondrial complex I, which is caused by peroxidative stress. |
| Stankiewicz and Brass (2009) Review | Investigation of the function of iron physiology in the brains of healthy adults and the relationship between increased iron deposition in the brain and common neurodegenerative diseases affecting the elderly. | Magnetic resonance imaging (MRI) of iron distribution in brain. | *In vivo* iron distribution in human brain (alive human); Animal model (mice) of different iron chelators. | Increased brain iron levels may induce neurotoxicity via free radical production, lipid peroxidation, and cell death. |
| Stankiewicz *et al.* (2007) Review | Focus on the imaging of brain iron and the underlying physiology and metabolism relating to iron deposition. | MRI is used to quantify *in vivo* iron measurement; Histological technique. | Rat and mouse models of PD; Rhesus monkey; Human brain. | Brain iron overload can cause free radical damage, lipid peroxidation, and cell death. This excessive iron can be potentially linked to many chronic brain disorders including Alzheimer's disease, PD, and multiple sclerosis. |

6-OHDA - 6-hydroxydopamine; α-KGDHC - alpha-ketoglutarate dehydrogenase complex; AA - ascorbic acid; ATP - adenosine triphosphate; Au - aurem/gold; Bax - B-cell lymphoma (Bcl)-2–associated X protein; Bim - Bcl-2–interacting mediator of cell death; BSA - bovine serum albumin; DA - dopamine; EGCG - epigallocatechin-3-gallate; ETCs - electron transfer complexes; FET - fluorescence energy transfer; HCT116 - human colon cancer cell line; HEK293T - human embryonic cancer cell line; HeLa - human cervical cancer cell line; L-cys ZnS:Mn QDs - L-cysteine-capped manganese-doped zinc sulphide quantum dots; Mn:ZnS/BSA QDs - BSA-capped manganese-doped zinc sulphide quantum dots; MPP+ - 1-methyl-4-phenylpyridinium ion; MPTP - 1-methyl-4-phenyl-1,2,3,6-tetrahydropyridine; MRI - magnetic resonance imaging; NADH – reduced nicotinamide adenine dinucleotide; NO - nitric oxide; NTA - nitrilotriacetic acid; PD - Parkinson's disease; PINK1 - phosphatase and tensin homolog (PTEN)-induced kinase 1; ROS - reactive oxygen species; SDHB - succinate dehydrogenase subunit B; SH-SY5Y - human neuroblastoma cell line; SN - substantia nigra; ssDNA - single-stranded DNA; STM - scanning tunneling microscopy; TIMMDC1 - translocase of inner mitochondrial membrane domain containing 1; TIQ – tetrahydroisoquinoline; UA - uric acid.

**References**

**Abha K, Sumithra IS, Suji S, Anjana RR, Anjali Devi JS, Nebu J, Lekha GM, Aparna RS & George S.** **2020.** Dopamine-induced photoluminescence quenching of bovine serum albumin–capped manganese-doped zinc sulphide quantum dots. *Analytical and Bioanalytical Chemistry* **412(23)**, 5671-5681. DOI 10.1007/s00216-020-02787-2

**Atta NF, Galal A & El-Said DM.** **2019.** Novel design of a layered electrochemical dopamine sensor in real samples based on gold nanoparticles/β-cyclodextrin/nafion-modified gold electrode. *ACS Omega* **4(19)**, 17947-17955. DOI 10.1021/acsomega.9b01222

**Benecke R, Strümper P & Weiss H.** **1993.** Electron transfer complexes I and IV of platelets are abnormal in Parkinson's disease but normal in Parkinson-plus syndromes. *Brain* **116(6)**, 1451-1463. DOI 10.1093/brain/116.6.1451

**Benzi G & Moretti A.** **1995.** Age- and peroxidative stress-related modifications of the cerebral enzymatic activities linked to mitochondria and the glutathione system. *Free Radical Biology and Medicine* **19(1)**, 77-101. DOI 10.1016/0891-5849(94)00244-E

**Camargo AF, Chioda MM, Rodrigues APC, Garcia GS, McKinney EA, Jacobs HT & Oliveira MT.** **2018.** Xenotopic expression of alternative electron transport enzymes in animal mitochondria and their impact in health and disease. *Cell Biology International* **42(6)**, 664-669. DOI 10.1002/cbin.10943

**Carreras MaC, Franco MaC, Peralta JG & Poderoso JJ.** **2004.** Nitric oxide, complex I, and the modulation of mitochondrial reactive species in biology and disease. *Molecular Aspects of Medicine* **25(1)**, 125-139. DOI 10.1016/j.mam.2004.02.014

**Chatterjee S, Ghosh S, Mishra S, Das Saha K, Banerji B & Chattopadhyay K.** **2019.** Efficient detection of early events of α-synuclein aggregation using a cysteine specific hybrid scaffold. *Biochemistry* **58(8)**, 1109-1119. DOI 10.1021/acs.biochem.8b01161

**Diaz-Diestra D, Thapa B, Beltran-Huarac J, Weiner BR & Morell G.** **2017.** L-cysteine capped ZnS:Mn quantum dots for room-temperature detection of dopamine with high sensitivity and selectivity. *Biosensors and Bioelectronics* **87**, 693-700. DOI 10.1016/j.bios.2016.09.022

**Ehlich R & Hörber JKH.** **2009.** Molecular order affecting electron transport through ssDNA. *Ultramicroscopy* **109(8)**, 1074-1079. DOI 10.1016/j.ultramic.2009.03.044

**Fato R, Bergamini C, Leoni S, Strocchi P & Lenaz G.** **2008.** Generation of reactive oxygen species by mitochondrial complex I: Implications in neurodegeneration. *Neurochemical Research* **33(12)**, 2487-2501. DOI 10.1007/s11064-008-9747-0

**Greenamyre JT, Sherer TB, Betarbet R & Panov AV.** **2001.** Complex I and Parkinson's disease. *IUBMB Life* **52(3-5)**, 135-141. DOI 10.1080/15216540152845939

**Guarani V, Paulo J, Zhai B, Huttlin EL, Gygi SP & Harper JW.** **2014.** TIMMDC1/C3orf1 functions as a membrane-embedded mitochondrial complex I assembly factor through association with the MCIA complex. *Molecular and Cellular Biology* **34(5)**, 847-861. DOI 10.1128/MCB.01551-13

**Hashimoto M, Takeda A, Hsu LJ, Takenouchi T & Masliah E.** **1999.** Role of cytochrome c as a stimulator of α-synuclein aggregation in Lewy body disease. *Biological Chemistry* **274(41)**, 28849-28852. DOI 10.1074/jbc.274.41.28849

**Imai Y.** **2020.** PINK1-Parkin signaling in Parkinson’s disease: Lessons from Drosophila. *Neuroscience Research* **159**, 40-46. DOI 10.1016/j.neures.2020.01.016

**Jiang X, Li L, Ying Z, Pan C, Huang S, Li L, Dai M, Yan B, Li M, Jiang H, Chen S, Zhang Z & Wang X.** **2016.** A small molecule that protects the integrity of the electron transfer chain blocks the mitochondrial apoptotic pathway. *Molecular Cell* **63(2)**, 229-239. DOI 10.1016/j.molcel.2016.06.016

**Konijnenberg A, Ranica S, Narkiewicz J, Legname G, Grandori R, Sobott F & Natalello A.** **2016.** Opposite structural effects of epigallocatechin-3-gallate and dopamine binding to α-synuclein. *Analytical Chemistry* **88(17)**, 8468-8475. DOI 10.1021/acs.analchem.6b00731

**Kuter KZ, Olech Ł & Dencher NA.** **2019.** Increased energetic demand supported by mitochondrial electron transfer chain and astrocyte assistance is essential to maintain the compensatory ability of the dopaminergic neurons in an animal model of early Parkinson's disease. *Mitochondrion* **47**, 227-237. DOI 10.1016/j.mito.2018.12.002

**La M, Chen CD, Li H & Yang CY.** **2014.** Modulation of iron catalytic activity to dopamine oxidation through additional iron ligation. *International Journal of Electrochemical Science* **9(12)**, 6870-6879.

**Lee JC, Gray HB & Winkler JR.** **2005.** Tertiary contact formation in α-synuclein probed by electron transfer. *Journal of the American Chemical Society* **127(47)**, 16388-16389. DOI 10.1021/ja0561901

**Lee JC, Lai BT, Kozak JJ, Gray HB & Winkler JR.** **2007.** α-Synuclein tertiary contact dynamics. *Journal of Physical Chemistry B* **111(8)**, 2107-2112. DOI 10.1021/jp068604y

**McEwen GDGD, Chen F & Zhou A.** **2009.** Immobilization, hybridization, and oxidation of synthetic DNA on gold surface: Electron transfer investigated by electrochemistry and scanning tunneling microscopy. *Analytica Chimica Acta* **643(1-2)**, 26-37. DOI 10.1016/j.aca.2009.03.050

**Mizuno Y, Ikebe S-i, Hattori N, Mochizuki H, Nakagawa-Hattori Y & Kondo T.** **1994.** Studies on the pathogenesis of Parkinson's disease in Japan. *Archives of Gerontology and Geriatrics* **19(2)**, 105-121. DOI 10.1016/0167-4943(94)90033-7

**Mizuno Y, Ikebe S-i, Hattori N, Nakagawa-Hattori Y, Mochizuki H, Tanaka M & Ozawa T.** **1995.** Role of mitochondria in the etiology and pathogenesis of Parkinson's disease. *Biochimica et Biophysica Acta (BBA) - Molecular Basis of Disease* **1271(1)**, 265-274. DOI 10.1016/0925-4439(95)00038-6

**Morikawa N, Naoi M, Maruyama W, Ohta S, Kotake Y, Kawai H, Niwa T, Dostert P & Mizuno Y.** **1998.** Effects of various tetrahydroisoquinoline derivatives on mitochondrial respiration and the electron transfer complexes. *Journal of Neural Transmission* **105(6-7)**, 677-688. DOI 10.1007/s007020050087

**Navarro A & Boveris A.** **2010.** Brain mitochondrial dysfunction in aging, neurodegeneration, and Parkinson's disease. *Frontiers in Aging Neuroscience* **2(SEP)**. DOI 10.3389/fnagi.2010.00034

**Naydenov AV, Vassoler F, Luksik AS, Kaczmarska J & Konradi C.** **2010.** Mitochondrial abnormalities in the putamen in Parkinson's disease dyskinesia. *Acta Neuropathologica* **120(5)**, 623-631. DOI 10.1007/s00401-010-0740-8

**Núñez MT, Urrutia P, Mena N, Aguirre P, Tapia V & Salazar J.** **2012.** Iron toxicity in neurodegeneration. *BioMetals* **25(4)**, 761-776. DOI 10.1007/s10534-012-9523-0

**Pastoris O, Dossena M, Foppa P, Catapano M, Ferrari R & Dagani F.** **1995.** Biochemical evaluations in skeletal muscles of primates with MPTP parkinson-like syndrome. *Pharmacological Research* **31(6)**, 361-369. DOI 10.1016/1043-6618(95)80090-5

**Stankiewicz J, Panter SS, Neema M, Arora A, Batt CE & Bakshi R.** **2007.** Iron in Chronic Brain Disorders: Imaging and Neurotherapeutic Implications. *Neurotherapeutics: The Journal of the American Society for Experimental NeuroTherapeutics* **4(3)**, 371-386. DOI 10.1016/j.nurt.2007.05.006

**Stankiewicz JM & Brass SD.** **2009.** Role of iron in neurotoxicity: A cause for concern in the elderly? *Current Opinion in Clinical Nutrition and Metabolic Care* **12(1)**, 22-29. DOI 10.1097/MCO.0b013e32831ba07c
